# Modelling the Mass Transfer Process of Malvidin-3-Glucoside during Simulated Extraction from Fresh Grape Solids under Wine-Like Conditions

**DOI:** 10.3390/molecules23092159

**Published:** 2018-08-27

**Authors:** Patrick C. Setford, David W. Jeffery, Paul R. Grbin, Richard A. Muhlack

**Affiliations:** 1 Department of Wine and Food Science, School of Agriculture, Food and Wine, The University of Adelaide, PMB 1, Glen Osmond SA 5064, Australia; patrick.setford@adelaide.edu.au (P.C.S.); david.jeffery@adelaide.edu.au (D.W.J.); paul.grbin@adelaide.edu.au (P.R.G.); 2 The Australian Research Council Training Centre for Innovative Wine Production, The University of Adelaide, PMB 1, Glen Osmond SA 5064, Australia

**Keywords:** phenolic extraction, diffusion, anthocyanin, process modelling, wine colour, mass transfer

## Abstract

Extraction of grape components is a key consideration for red winemaking. The impact of changing process variables on mass transfer properties of anthocyanins from fresh pre-fermentative red grape solids under forced convective conditions was explored using the dominant red grape anthocyanin, malvidin-3-glucoside (M3G) as a model solute. A two level full factorial design was implemented to investigate effects of temperature, sugar and ethanol on mass transfer properties. Factor levels were chosen to simulate conditions found at various points during the maceration and fermentation steps of the red winemaking process. A rigorous mathematical model was developed and applied to experimental extraction curves, allowing the separation of mass transport properties in liquid and solid phases in a wine-like system, for the first time. In all cases, the coefficient of determination exceeded 0.92, indicating good agreement between experimental and mathematically-solved M3G concentrations. For the conditions studied, internal mass transfer was found to limit M3G extraction and changes to the liquid phase composition and temperature influence the distribution constant. Surface response models of mass transfer parameters were developed to allow future simulations of fermentation scenarios aimed at maximising the extraction potential of M3G.

## 1. Introduction

Malvidin-3-*O*-β-d-glucoside (M3G) is frequently the focus of red wine research due to its relatively high importance to colour (including derived pigments) and overall quality. Understanding the effect of physical and chemical parameters on the extraction and subsequent evolution of phenolic compounds, and in particular anthocyanins, is crucial for producing red wine of high quality with desired sensorial characteristics.

In traditional red winemaking the grape solids typically remain in contact with the juice for around one to two weeks, from the time of grape crushing and well into the fermentation period of the must (fermenting juice and solids). During this time, anthocyanins are extracted from the semi-porous skins via the mechanism of solid-liquid diffusion, where the concentration slowly accumulates in the liquid over a period of several days until the rate of accumulation of monomeric anthocyanins is exceeded by the rate of subsequent reactions, including condensation, self-association, co-pigmentation, oxidation and physical adsorption processes with grape solids and yeast lees [1]. As a consequence of fermentative maceration, the upward force from evolving carbon dioxide raises the grape solids to the top of the fermenting vessel, forming a cap which limits both the solid contact area with the bulk of the liquid and the phenolic potential of the wine. Winemakers typically adopt one of several methods to mix the solids and increase the solid-liquid contact area to help facilitate phenolic (including anthocyanin) extraction. Methods typically include either mechanically punching down the solids into the liquid to break up the cap, pumping over liquid from the bottom of the tank and spraying it over the top to resubmerge the cap, or fermenting in a baffled rotary tank that periodically revolves and submerges the solids into the liquid. Despite this, conflicting results on the method of contacting with respect to phenolic extraction potential have been found [1], indicating that solute diffusion within the solid to the solid-liquid interface may be limiting.

In the past, methods of enhancing contact between the solid and liquid phases of grape must have previously been qualitatively compared for different grape varieties [2,3]. To the authors’ knowledge however, there have been no reported attempts at modelling these physical processes and systems to define what is happening at the solid-liquid interface so that the different extraction techniques can be systematically compared. Previously, simple correlations have been derived to model the extraction and subsequent evolution of anthocyanins and other phenolic compounds during red wine maceration and fermentation [4,5,6]. These forms of kinetic models can be used to provide information and insight into the impact of changing process conditions and are very effective at fitting experimental data. However, there is limited ability to quantitatively predict the effect of mixing (maceration) operations or changing process conditions during fermentation, such as temperature and liquid phase concentrations of ethanol, sugar, and water on the extraction kinetics and final equilibrium concentrations of phenolics. In contrast, empirical correlations in conjunction with systems of dimensionless numbers describing the fluid flow have been widely used in other biological systems to estimate the external diffusion coefficient of phenolic compounds. This method has been used in both semi-batch [7,8] and batch [9,10,11] solid-liquid extraction systems to determine the effect of mixing on the rate of phenolic extraction. This approach has not hitherto been applied to a red wine fermentation system.

This study sought to evaluate the mass transfer and diffusive properties of M3G as an important model solute during solid-liquid extraction from fresh red grape solids under forced convective conditions at various simulated stages of red wine fermentation with regards to varying solvent and temperature conditions.

## 2. Results and Discussion

### 2.1. Extraction Kinetics and Model Analysis

**Figure 1:**
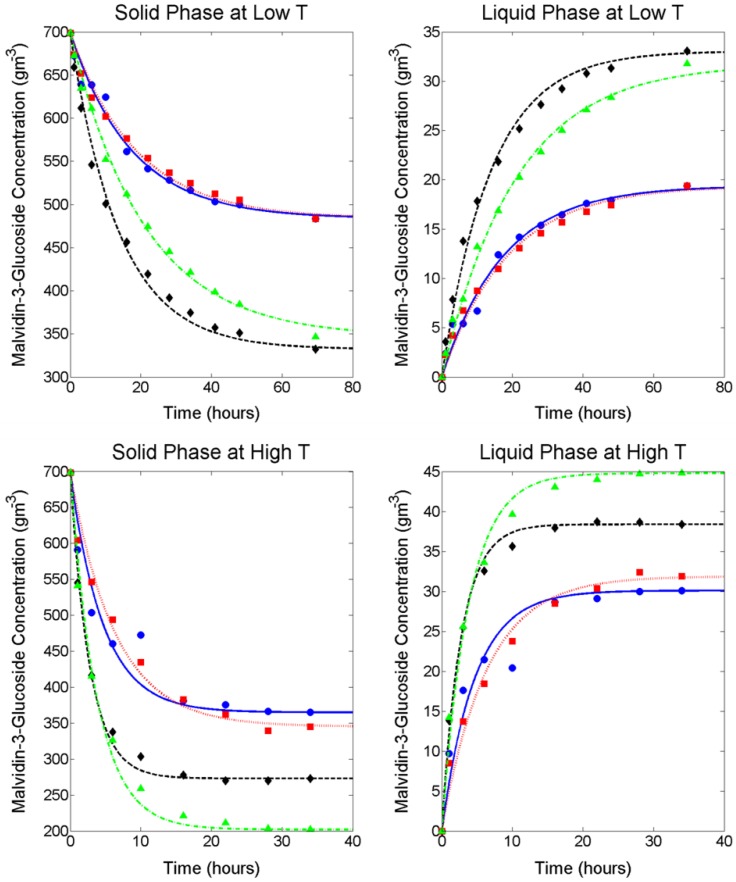
Experimental and fitted models for malvidin-3-glucoside solid phase depletion (**left**) and liquid phase accumulation (**right**) at low temperature (**upper**) and high temperature (**lower**) conditions. ●, water; ■, 266 g/L glucose; ◆, 14% *v*/*v* ethanol; ▲, 266 g/L glucose and 14% *v*/*v* ethanol.

**Figure 2:**
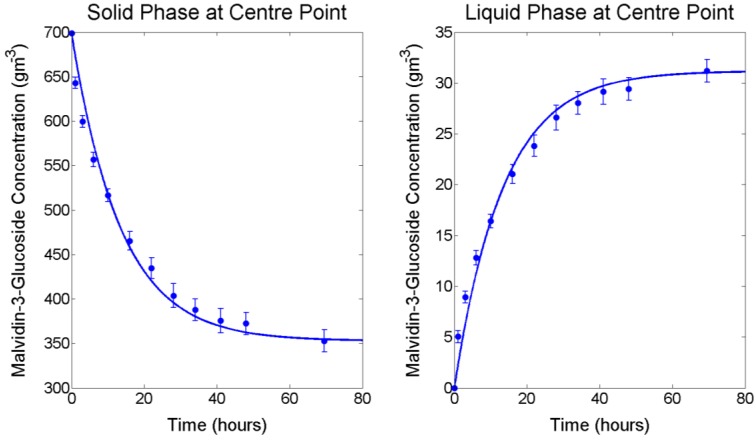
Experimental and fitted model for malvidin-3-glucoside solid phase depletion (**left**) and liquid phase accumulation (**right**) at the factorial centre point conditions (12.2 °C, 133 g/L glucose and 7% ethanol). Error bars represent standard deviation.

M3G extraction from fresh pre-fermentative Merlot grape pomace was examined using a 2^3^ full factorial design under forced convective conditions with solutions differing in temperature, and sugar and ethanol concentrations to simulate fermentative extraction. Although forced convective conditions are usually only present during fermentative maceration during mechanical mixing operations (with the exception of the mixing effect caused by CO_2_ evolution and temperature stratification), constant mixing was employed for this experiment so that the liquid phase could be considered a homogenous mixture without an anthocyanin concentration gradient. This allowed for the mass transfer properties of internal and external diffusion to be evaluated independently from one another and to provide an accurate evaluation of the extractive behaviour within the grape solids under the chosen experimental conditions. For all factor combinations, mean values of solid and liquid phase extraction and accumulation of M3G throughout maceration together with the model solution using the method described in Section 3.2 and Section 3.3 are presented in Figure 1 and the mid-point is presented in Figure 2. For low- and mid-temperature levels (4.4 °C and 12.2 °C respectively), the concentration of M3G in the liquid phase increased throughout the extraction period, whereas at the high temperature level (23.1 °C) the concentration began to decrease after reaching a maximum. Because this work is concerned with modelling the extraction of M3G and the majority of anthocyanin extraction takes place within the first days of liquid contacting, experimental data up to and including the maximum concentration in the liquid phase was used for modelling the extraction process according to Fick’s second law.

A summary of the internal and external diffusion and mass transfer coefficients, as well as several relevant mass transfer properties calculated are presented for each experimental condition in Table 1, together with $R^{2}$ and *RMSE* $R^{2}$ values were >0.92 and small *RMSE* values were observed in all cases. Inclusion of a replicated centre point within the full factorial design provided an estimate of standard error (ranging from 0.29 to 0.62 g m^−3^) and repeatability (coefficient of variation (CV) of 0.12 for the initial 1 h extraction sample, followed by CV values ranging from 0.036 to 0.067) throughout the extraction process. Regression residuals are consistent with this standard error range, indicating the model developed and presented in Section 3.2 and Section 3.3 can be used to effectively predict M3G extraction rates from fresh grape solids at conditions simulating various stages of the fermentation process.

Complete ANOVA tables for $D_{s\beta }$, $k_{c\beta }$ and K are provided in Tables S1–S3 respectively. As evidenced from the factorial analysis (presented in Table 2) and Figure 1, the inclusion of ethanol in the liquid phase had a positive influence on both the rate of extraction ($k_{c\beta }$) as well as the final concentration of M3G (K) at all temperature conditions (p < 0.05), although glucose was not found to significantly affect internal diffusion ($D_{s\beta }$) and mass transfer ($k_{c\beta }$). This may be due to ethanol improving the solvent properties of the penetrating liquid allowing easier dissolution of anthocyanins. The main effect of glucose on K is significant (p < 0.01), however the factor effect is approximately 70% smaller than that of either temperature or ethanol. Therefore, at low temperature conditions, it appears that the inclusion of glucose in the liquid phase had only a minor influence on the final concentration of M3G when compared to the pure water extracts and 14% ethanol extracts, respectively. In contrast, at high temperature conditions, the inclusion of both glucose and ethanol resulted in an increase in final concentration of M3G in the liquid phase when compared to the extract containing only 14% ethanol, with factorial analysis confirming temperature, glucose and ethanol effects on K were all significant (p < 0.05). This result indicates a previously unobserved phenomenon whereby at higher temperatures, glucose in the presence of ethanol appeared to aid the extraction rate and maximum extractability of M3G. Although interesting, the scenario of high sugar and high ethanol concentrations is unlikely to be observed in a typical red winemaking operation and was necessary only to fulfil the requirements of the experimental design. In agreement with other studies [12,13], increasing the temperature of the system had a large positive impact on both the rate and the final extraction yield of anthocyanins.

This result could be explained by the increased solubility and diffusivity of M3G at higher temperatures as well as an increased rate of swelling and softening of the solid material [12].

As shown in Table 1, internal solid diffusion rates obtained from the model solution are approximately one to two orders of magnitude smaller than their respective external diffusion rates in the liquid phase. This is expected, as internal diffusion encompasses liquid penetration, solute dissolution and the subsequent diffusion through the solid matrix, which is a tortuous diffusion path [10]. Furthermore, the internal mass transfer coefficients are six to seven times smaller in order of magnitude than the respective external mass transfer coefficients, giving a good indication that the extraction of M3G in this system is heavily dependent on diffusion within the solid. In general, diffusion within the solid is typically the rate-controlling step during solid-liquid extraction of phenolic compounds [13,14]. The extent of control can be numerically indicated as the mass analogue of the Biot number (full nomenclature and equation notation can be found in Appendix A, Appendix B, Appendix C and Appendix D):

(1) $$Bi=\frac{k_{c\gamma }LK}{D_{s\beta }}$$

A Biot number exceeding 10 indicates that internal diffusion within the solid is the controlling step of the extraction process. In the present study, values of Bi > 10^4^ were determined for all factor combinations, confirming that the internal diffusion within the solid was indeed the rate limiting step of the extraction process. Such high Biot numbers are due to the constant mixing and relatively high liquid velocity, whereas in a traditional red wine fermentation scenario this is unlikely to be the case. Mixing operations during fermentation are typically performed intermittently throughout the extraction process, and for the majority of the time, any mixing in the liquid phase would be the result of evolved CO_2_ displacing the liquid. Because of this, the internal mass transfer rates solved for each set of conditions in this study represent the maximum extraction rates possible for a real red wine fermentation and provide insight into the minimum time required to achieve the maximum potential M3G concentration.

Nonetheless, understanding the Biot number and how it can be manipulated under different conditions could be used in the targeted development of mixing technology to optimise the mass transfer coefficient in the liquid phase. This could be accomplished by controlling the fluid velocity in a way that does not compromise the quality of the wine through increased oxidation nor interfere with downstream processing through the potentially increased destruction of grape solids (primarily skins) resulting from vigorous extraction procedures and mixing operations. Schmidt and Velten [15] found the average velocity of the liquid phase at the most active stages of wine fermentation to be approximately 0.21–0.60 m s^−1^. At the lower end of these velocities and where the effects of mixing due to fermentation are minimal, extraction could be such that internal diffusion is no longer the rate-determining step in the extraction of M3G (and other phenolic compounds). As such, an interesting area for further study would be to examine the effects of free convective conditions at various stages of red wine fermentation in order to calculate external mass transfer coefficients and gain further insight into the importance of mixing operations, their induced liquid velocities, the timing of mixing, and the length of time required to adequately facilitate the extraction process. Such insight would be of additional value due to the compressible nature of grape solids, whereby the upward force of evolving carbon dioxide during fermentative maceration results in a separation of the solids with the bulk of the liquid. This could be considered to be causing an additional liquid phase mass transfer step, as the solute at the solid-liquid interface must first diffuse into the interstitial liquid within the skin cap before making its way into the liquid bulk via liquid phase diffusion through the cap or by forced convection caused by mixing operations.

In agreement with other studies that model the extraction of anthocyanins from various biological materials [7,9,13], the internal diffusion and mass transfer coefficients were found to be higher at higher temperatures, and the inclusion of ethanol in the liquid phase at wine-relevant concentrations was also found to promote the rate of internal diffusion. For each set of solvent conditions, the distribution constant K at high temperature is found to be approximately double that found at the respective low temperature (shown in Table 1). Because the distribution constant is a linear function of the solid-liquid ratio (as shown in Equation (8)), the values found at each condition could be used to either help maximise the concentration of M3G or optimise it towards a specific desired concentration in red wine ferments that will maximise the perceived quality of the finished product. Notably, the lower distribution constant values found in this study at low ethanol concentrations (Table 1) provide evidence that maceration techniques prior to fermentation (particularly when conducted at colder temperatures) may have little impact on the final concentration of anthocyanins after the skins are removed from the wine, and that extended maceration upon the completion of fermentation, where a higher concentration of ethanol is in the liquid phase, is more likely to increase the final anthocyanin concentration. This finding also helps to explain experimental results from other studies such as that by Koyama et al. [16], who found that undertaking a cold soaking operation prior to the fermentation of red wine resulted in a slower initial extraction rate of anthocyanins and proanthocyanidins but has no significant effect on the maximum concentrations of these phenolic compounds at the end of a 10-day maceration period.

### 2.2. Response Surface Analysis

**Figure 3:**
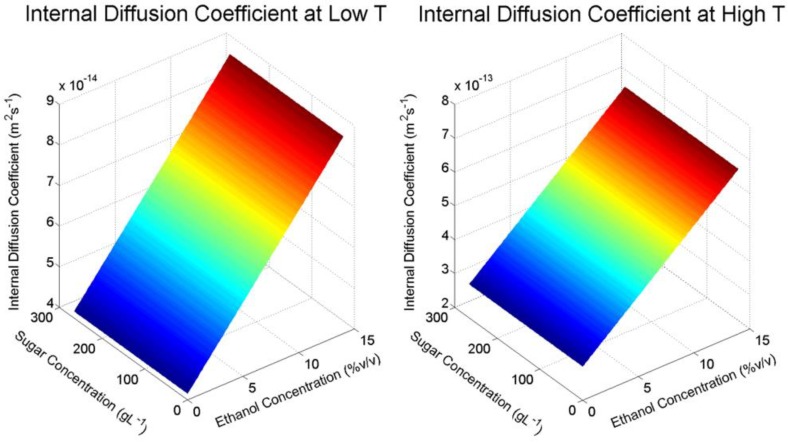

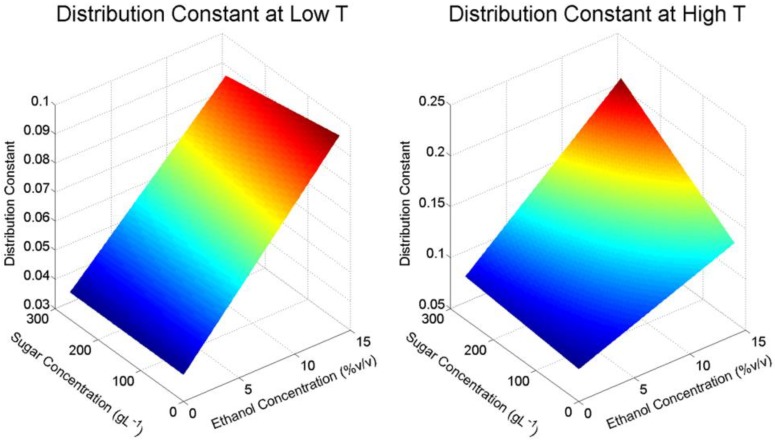
Surface response of malvidin-3-glucoside internal diffusion coefficient, $k_{c\beta }$ (**upper**) and distribution constant, K (**lower**) at changing solvent conditions for low temperature (**left**) and high temperature (**right**) in the factorial design.

From the design of the experiment and the applied statistical analysis, expressions were generated that allowed for the estimation of the internal diffusion coefficient ($D_{s\beta }$) and distribution constant (K) within the range of experimental conditions using statistically significant variables:

(2) $$D_{s\beta }=2.75\times 10^{-13}+2.11\times 10^{-13}T+1.09\times 10^{-16}C_{EtOH}+8.52\times 10^{-14}TC_{EtOH}$$

(3) $$\begin{array}{cc} K=9.97\times 10^{-2} & +3.33\times 10^{-2}T+1.04\times 10^{-2}C_{g}+3.74\times 10^{-2}C_{EtOH}\\ & +1.23\times 10^{-2}TC_{g}-9.64\times 10^{-3}TC_{EtOH}+7.91\times 10^{-3}C_{g}C_{EtOH}\\ & +9.86\times 10^{-3}TC_{g}C_{EtOH} \end{array}$$

where T is the temperature (°C), $C_{g}$ is the concentration of sugar in the liquid phase (g L^−1^) and $C_{EtOH}$ is the concentration of ethanol in the liquid phase (% *v*/*v*) scaled in terms of the coded variables used in the ANOVA (−1 for low, 0 for the midpoint and 1 for the high values, respectively). Graphical representations of the resulting response surfaces are presented in Figure 3, where the effect of ethanol and glucose concentrations at the low and high temperature conditions used for the factorial design are shown. From Figure 3, a clear increase in $D_{s\beta }$ with increasing ethanol concentration can be seen, as well as an order of magnitude increase in $D_{s\beta }$ from low to high temperature. From the analysis of variance in Table 2, it can be seen that the factor effect of glucose on internal diffusion is negative, however the overall significance across the range of the factorial experiment is negligible (p > 0.05).

### 2.3. Application

In recent years, several studies have highlighted a lack of knowledge regarding the kinetics and mechanisms of phenolic extraction during red wine fermentation [1,17]. Due to this absence of understanding and despite the crucial role of phenolics to wine quality, a distinct difficulty exists with the informed manipulation of the phenolic content during the fermentative maceration phase. The method developed in this study to model the extraction of M3G from fresh grape solids under simulated red wine processing conditions allows for the separation of the mass transfer properties in the solid and liquid phases respectively—An important distinction from studies that have previously modelled extraction during fermentation. The current method gives greater insight into the extractive behaviour of M3G under different process conditions simulating those found during real winemaking scenarios. In doing this, the mass transfer properties determined in this study could be used to simulate the extraction of M3G under different conditions and various fermentation scenarios where the liquid phase is a medium with continuously changing ethanol and sugar concentrations. In this way, simulations using the presented mass transfer coefficients and distribution constants could be used to inform winemakers of optimal temperature conditions, the extent and time of fermentative mixing operations, and the time of pressing from skins to achieve a desired extraction rate or final anthocyanin concentration, prior to commencing a real-time fermentation. Additionally, the ability to predict and simulate extraction scenarios could be used to inform the targeted development of mixing technologies that include the optimisation of velocity in a way that maximises the external mass transfer and extraction potential without compromising the quality of the finished product or the ability to manage the grape solids in downstream processes.

## 3. Materials and Methods

### 3.1. Experimental

#### 3.1.1. Experimental Design

A 2^3^ full factorial design run in duplicate with centre point run in quadruplicate was used to investigate the effect of temperature, ethanol concentration, sugar (glucose) concentration, and their interactions, on the rate and extent of M3G extraction. Ethanol and glucose were added to water at low and high level concentrations chosen to emulate conditions that would be found in unfermented juice (0% *v*/*v* ethanol and 266 g L^–1^ sugar) and in the equivalent finished red wine (14% *v*/*v* ethanol and 0 g L^–1^ sugar). Other chemical compounds typically found in wine were not included in order to understand the direct impact of the two largest components of red grade juice and wine responsible for the physical properties of the liquid, thus limiting confounding variables. As such, pH values were not adjusted but were determined to be 3.7 and 3.9 after one hour of extraction. Low and high extraction temperatures nominally set at 0 °C and 20 °C were chosen to investigate the impact on extraction rate during fermentation as well as to allow for conclusions to be drawn regarding extraction during low temperature processes common in commercial winemaking such as pre-fermentative cold soaking. Real-time data logging of the winery cold rooms used for this investigation showed ambient air temperatures of 4.4 °C and 23.1 °C, respectively, with a mid-point temperature of 12.2 °C. As such, these temperatures were used for the purposes of mathematical modelling and subsequent generation of results as they represent realistic industry conditions.

#### 3.1.2. Sample Preparation

Fresh Merlot grapes were hand harvested (total soluble solids of 14.3° Baumé, pH 3.6, titratable acidity (to pH 8.2) of 3.9 g L^–1^) on 24 February 2016 and kept for 7 days in a 0 °C cool room until crushing. Berries were destemmed by hand and the total weight prior to crushing was recorded. A total of 9.05 kg of randomly sampled berries were then crushed by hand using a hand plunger until all berries were visibly crushed and no whole berries remained intact. The must was then pressed using a 4.4 L, hand operated, stainless steel basket press to separate the solids (skins and seeds) and liquid juice. The total mass of pressed solids was 4.00 kg and the volume of juice was 4.55 L. giving a solid to liquid ratio of 0.88 kg L^–1^.

#### 3.1.3. Extraction Procedure

Portions of the pressed solids (150 g of skins and seeds) were placed into 2 L containers and 1.5 L of extraction solvent (as described in Section 3.1.1) were then added to begin the extraction process. A lower solid/liquid ratio than found in a typical red wine fermentation was chosen for the extraction procedure in order to limit the reaction rate between anthocyanins and other extracted compounds that could impact the perceived extraction rate. These solvents were prepared 48 h prior to the commencement of extraction and placed into the respective 0, 10 and 20 °C cool rooms to equilibrate to the ambient air temperature. Upon the addition of the liquid phase, lids were placed on the vessels with each having an approximately 1 cm diameter hole to allow insertion of the shaft of an overhead mechanical stirrer. The vessel contents were continually stirred at a speed of approximately 300 rpm to ensure the system was well-mixed and to allow for the calculation of mass transfer variables within the liquid phase. Samples (10 mL) were taken throughout the extraction process until an equilibrium was reached, with samples taken more frequently within the first 12 h of the extraction so that appropriate concentration curves could be generated. Samples were immediately centrifuged at 3220 rcf for 10 min and the supernatant decanted and kept at −20 °C until their analysis by HPLC. Solid phase M3G concentrations were calculated via mass balance based on the initial concentration in the solids.

#### 3.1.4. Quantification of Malvidin-3-Glucoside

Prior to HPLC analysis, samples were defrosted at ambient conditions and 2 mL of sample were then centrifuged at 9300 rcf to remove any suspended solids. 1.5 mL of supernatant was then transferred to amber HPLC vials for analysis. HPLC analyses were performed using an Agilent 1100 Instrument (Agilent, Forest Hill, VIC, Australia) equipped with a quaternary pump and diode array detector (DAD), using gradient elution based on the method described by Cozzolino et al. [18] with a slight modification as detailed by Mercurio et al. [19]. Data acquisition and processing were performed using Agilent ChemStation software (version B.01.03). A 20 µL injection volume was used for each sample, and chromatograms were recorded at 280, 320, 370 and 520 nm. M3G was quantified at 520 nm by comparison of its retention time and absorbance using malvidin-3-*O*-glucoside chloride (≥95% by HPLC) purchased from Extrasynthese (Genay, France) and prepared volumetrically in 0.2% aqueous acetic acid as an external standard.

#### 3.1.5. Measurement of Total Malvidin-3-Glucoside Concentration in Solids

Triplicate samples of 250 g of the freshly pressed grape solids were homogenised using a Grindomix GM200 homogeniser (Retsch, Haan, Germany) at a speed of 8000 rpm for 20 seconds. The homogenate was then well mixed and approximately 1 g was transferred to a 10 mL centrifuge tube, 10 mL of 50% *v*/*v* aqueous ethanol was added and the mixture was inverted every 10 min by hand throughout a one hour extraction period. The mixture was then centrifuged at 3220 rcf for 10 min and the supernatant transferred to a new centrifuged tube and frozen at −20 °C until HPLC analysis as described above (method adapted from Mercurio et al. [19]). Prior to injection, these samples were diluted to 12.5% *v*/*v* aqueous ethanol in water.

#### 3.1.6. Measurement of Physical Parameters

A Viscoball Höppler viscometer (Fungilab, Barcelona, Spain) was used to measure the viscosity of the extraction solutions at the extraction temperatures used during this experiment. Cameras (model KYT-U130-01MBWCS, Kayeton Technology Company Ltd, Shenzhen, China) at a frame rate of 30 frames per second were used to monitor the start and end points of the viscometer in order to increase the precision of time measurements.

### 3.2. Model Development

During the maceration stage of winemaking, anthocyanins compounds from the grape skins are extracted by two main mechanisms. The first of these is a fast leakage that occurs at the edges of broken skin cells as a result of crushing, followed by diffusion across the solid layers in a direction perpendicular to the surface of the skin towards the interior of the berry for the remainder of the solid-liquid contact period [1,20,21]. In general, the solid-liquid extraction of phenolic compounds from porous solid particles is a concentration-driven process that involves three steps which encapsulate internal diffusion. These are:

(1) Solvent diffusion into the porous solid
(2) Solute dissolution into the solvent
(3) Dissolved solute diffusion to the particle surface

From here, a final mass transfer step takes place whereby the dissolved solute must then travel from the particle surface to the surrounding solvent, either through free convective diffusion or through forced convective mixing [1,13,14]. Because grape skins have an outer waxy layer that impedes liquid penetration (and thus extraction of anthocyanins and other phenolics) it was assumed that after crushing, diffusion within the solid occurred primarily along the perpendicular axis to the main face of the skin in a single direction across the whole thickness of the skin (L), which can be considered a plane surface. As such, Fick’s second law can be applied to describe diffusion within the solid phase:

(4) $$\frac{\partial c_{\beta }}{\partial t}=D_{s\beta }\frac{\partial^{2}c_{\beta }}{\partial x^{2}}$$

where x is the distance co-ordinate in the direction of diffusion. Diffusion takes place only in a single direction (as opposed to a leaf for instance where diffusion would take place in both directions perpendicular to the face of the leaf) so the distance coordinate extends from x=0 to x=L.

For particles of a fixed geometry, previous studies [20,21] have demonstrated that the mass transfer by averaged effective diffusion within both phases can be represented as a macroscopic mass transfer system of ordinary differential equations—A typical simplification used in mass transfer operations [22] which has previously been used to model solid-liquid phenolic extraction in vanilla pods [10] and coffee beans [11]:

(5) $$\varepsilon \frac{dc_{\gamma }}{dt}=k_{c\gamma }a\left(c_{\gamma i}-c_{\gamma }\right)$$

(6) $$\left(1-\varepsilon \right)\frac{dc_{\beta }}{dt}=k_{c\beta }a\left(c_{\beta i}-c_{\beta }\right)$$

(7) $$k_{c\gamma }\left(c_{\gamma i}-c_{\gamma }\right)=-k_{c\beta }a\left(c_{\beta i}-c_{\beta }\right)$$

(8) $$c_{\gamma i}=Kc_{\beta i}$$

where Equation (8) presents a distribution constant allowing for the description of the equilibrium between phases.

In order to relate the microscopic and macroscopic mass transfer properties, an analytical solution of Fick’s second law is required. The solution to Equation (4) is an infinite Fourier series analogous to conductive heat transfer in 1-dimension:

(9) $$\begin{array}{c} \frac{c_{\beta }-c_{\beta i}}{c_{\beta 0}-c_{\beta i}}=\frac{4}{\pi }[\frac{1}{1}exp\left(-\frac{1^{2}\pi^{2}X}{4}\right)sin\frac{1\pi x}{2L}\\ +\frac{1}{3}exp\left(-\frac{3^{2}\pi^{2}X}{4}\right)sin\frac{3\pi x}{2L}+\frac{1}{5}exp\left(-\frac{5^{2}\pi^{2}X}{4}\right)sin\frac{5\pi x}{2L}+\ldots ] \end{array}$$

where:

(10) $$X=\frac{D_{s\beta }t}{L^{2}}$$

Using an approximation described by Stapley [23] and employed by others [10,11], the faster decaying terms of the summation can be neglected due to the fact that for sufficiently high values of t, these terms decay to a negligible amount (second and third terms decay 9 and 25 times faster than the first with a third and fifth of the amplitude, respectively), and the first term can be used to approximate the solution. Therefore, at the boundary layer where x=L:

(11) $$\frac{c_{\beta }-c_{\beta i}}{c_{\beta 0}-c_{\beta i}}=\frac{4}{\pi }exp\left(-\frac{\pi^{2}D_{s\beta }t}{4L^{2}}\right)$$

Rearranging Equation (11) and taking the derivative of $c_{\beta }$ with respect to t gives:

(12) $$\frac{dc_{\beta }}{dt}=\frac{\pi^{2}D_{s\beta }}{4L^{2}}\left(c_{\beta i}-c_{\beta }\right)$$

The simplified microscopic model, Equation (12), can now be compared with the macroscopic model, Equation (6), to yield the following equation relating internal diffusion and internal mass transfer:

(13) $$D_{s\beta }=\frac{k_{c\beta }4L}{\left(1-\varepsilon \right)\pi^{2}}$$

### 3.3. Parameter Estimation and Statistical Analysis

An analytical solution to the macroscopic model described by Equations (5) and (6) obtained by Laplace transformation was proposed by Espinoza-Pérez, Vargas, Robles-Olvera, Rodríguez-Jimenes and García-Alvarado [11] and yielded the following set of simultaneous equations describing the average concentration within both phases:

(14) $$c_{\beta }=c_{\beta 0}\left(C_{1}e^{r_{1}t}+C_{2}e^{r_{2}t}\right)$$

(15) $$c_{\gamma }=c_{\beta 0}\left(C_{3}e^{r_{1}t}+C_{4}e^{r_{2}t}\right)$$

where:

$$\begin{array}{c} r_{1,2}=-\frac{b_{1}+b_{2}}{2}\pm \frac{\sqrt{{\left(b_{1}+b_{2}\right)}^{2}-4\left(b_{1}b_{2}-b_{3}b_{4}\right)}}{2}\\ C_{1}=\frac{r_{1}+b_{1}}{r_{1}-r_{2}},C_{2}=\frac{r_{2}+b_{1}}{r_{2}-r_{1}},C_{3}=\frac{b_{3}}{r_{1}-r_{2}},C_{4}=\frac{b_{3}}{r_{2}-r_{1}}\\ b_{1}=\frac{k_{c\gamma }a\left(1-\psi_{1}\right)}{\varepsilon },b_{2}=\frac{k_{c\beta }a\left(1-\frac{\psi_{2}}{K}\right)}{\left(1-\varepsilon \right)},b_{3}=\frac{k_{c\gamma }a\psi_{2}}{\varepsilon },b_{4}=\frac{k_{c\beta }a\frac{\psi_{1}}{K}}{\left(1-\varepsilon \right)}\\ \psi_{1}=\frac{1}{1+\frac{k_{c\beta }}{Kk_{c\gamma }}},\;\psi_{2}=\frac{k_{c\beta }/k_{c\gamma }}{1+\frac{k_{c\beta }}{Kk_{c\gamma }}} \end{array}$$

In order to calculate the external mass transfer coefficient, the following system of dimensionless equations that describe the forced convective mass transfer around spheres was used [22]:

(16) Sh=2+0.95Re12Sc13

where the Reynolds number (Re) was estimated as a function of the mixer diameter (D) and revolutionary speed (N) in revolutions per second:

(17) $$Re=\frac{ND^{2}\rho_{\gamma }}{\mu_{\gamma }},\;Sh=\frac{k_{c\gamma }L}{D_{s\gamma }},\;Sc=\frac{\mu_{\gamma }}{\rho_{\gamma }D_{s\gamma }}$$

In order to obtain the external diffusion coefficient ($D_{s\gamma })$ required to calculate the Schmidt and Sherwood numbers and thus solve the dimensionless system, the Wilke-Chang correlation was used:

(18) $$D_{s\gamma }=\frac{1.173\times 10^{-16}{\left(\varphi M_{\gamma }\right)}^{1/2}T}{\mu_{\gamma }V_{A}^{0.6}}$$

The molar volume of the solute ($V_{A})$ was estimated using an additive method described by Geankoplis [22] based on the chemical structure of M3G, and the average molecular mass and association factor of the liquid phases were estimated based on the mole fractions of ethanol, water and glucose in each trial:

(19) $$M_{\gamma }=x_{EtOH}M_{EtOH}+x_{water}M_{water}+x_{Glucose}M_{Glucose}$$

(20) $$\varphi =x_{EtOH}\varphi_{EtOH}+x_{water}\varphi_{water}+x_{Glucose}\varphi_{Glucose}$$

Finally, the internal mass transfer coefficient of M3G ($k_{c\beta }$) was obtained through a non-linear regression of Equations (14) and (15) using MATLAB software (version R2013a), whereby the residuals of the model solution were set to be minimised. A summary of the physical parameters, system variables and their method for determination is presented in Table 3.

In order to determine the efficacy of the proposed model’s fit with respect to experimental data, two parameters, the coefficient of determination ($R^{2}$) and the root mean square error (RMSE), were analysed for each set of experimental conditions. The coefficient of determination and root mean square error were determined by:

(21) $$R^{2}=1-\frac{\sum_{i=1}^{N}{\left(c_{\gamma ,pred,i}-c_{\gamma ,exp,i}\right)}^{2}}{\sum_{i=1}^{N}{\left(c_{\gamma ,pred,i}-{\bar{c}}_{\gamma ,exp}\right)}^{2}}$$

(22) $$RMSE=\sqrt{\frac{1}{N}\overset{N}{\underset{i=1}{\sum }}{\left(c_{\gamma ,pred,i}-c_{\gamma ,exp,i}\right)}^{2}}$$

where $c_{\gamma ,exp,i}$ is the experimentally determined concentration in the liquid phase, $c_{\gamma ,pred,i}$ is the concentration in the liquid phase predicted by the model, ${\bar{c}}_{\gamma ,exp}$ is the mean value of the experimentally-determined concentrations in the liquid phase and *N* is the number of data points for each experimental condition. The influence of changing experimental conditions was assessed for the internal diffusion coefficient ($D_{s\beta }$), external mass transfer coefficient ($k_{c\gamma }$) and distribution constant (K) by the analysis of variance (ANOVA). The significance of each parameter was determined by the corresponding p values, where values of p < 0.05 were deemed significant.

The inclusion of a centre point in the experimental design allowed for the development of a response surface (R) to be generated, where values of the internal diffusion coefficient and distribution constant were solved at each set of conditions were used to minimise the sum of squared residuals:

(23) $$R=b_{0}+b_{1}T+b_{2}C_{g}+b_{3}C_{EtOH}+b_{4}TC_{g}+b_{5}TC_{EtOH}+b_{6}C_{g}C_{EtOH}+b_{7}TC_{g}C_{EtOH}$$

where $b_{0}$ is the value of the function at the centre point conditions $b_{1}$, $b_{2}$, $b_{3}$ represent the effects of individual parameters associated to their respective variable, and $b_{4}$, $b_{5}$, $b_{6}$ and $b_{7}$ represent the crossed effects between variables. A clear distinction should be noted between the response surface equation and that which would be generated from the main effects in the ANOVA analysis, as the response equation is calculated by minimising the mean square error and includes a centre point to improve the model fit and thus the predictive capacity of response variables at points within the range of the experimental design. Furthermore, the inclusion of a centre point in the experimental design allowed for the calculation of standard error and standard deviation for this set of experimental conditions, which gave insight into the repeatability of the extractions across the experimental design.

## 4. Conclusions

Extraction curves showing M3G extraction from fresh Merlot grape solids under simulated red wine processing conditions have been reported and analysed. Rigorous mass transfer equations based on first principles and empirical correlations that describe this process were used to develop a complete mathematical model capable of accounting for both diffusion within the solid phase and the effect of mixing. The $R^{2}$ values of the proposed model indicated that it can be reliably used to predict M3G extraction through the calculation of internal and external mass transfer coefficients. In this study, the limiting factor for M3G extraction was found to be internal mass transfer while changes to the temperature and liquid phase composition directly impacted the distribution constant (and thus the maximum extractability). The design of the experiment allowed for a response surface equation to be developed that is capable of predicting the internal diffusion coefficient and distribution constant at conditions not experimentally evaluated. The development of these equations will allow for future computer simulation case studies to be conducted in order to predict the extraction of M3G (or other phenolics) during an active red wine fermentation scenario with continuously changing liquid phase conditions.

## Figures and Tables

**Table 1:** Summary of mass transfer properties ($D_{s\gamma }$, $k_{c\gamma }$, $D_{s\beta }$, $k_{c\beta }$ and K), Biot numbers (Bi) and statistical parameters (RMSE and $R^{2}$) for malvidin-3-glucoside solved using the method outlined in Section 3.2 and Section 3.3.

Trial Conditions			Diffusion and Mass Transfer Properties						Model Fit	
Temp.	Glucose	Ethanol	*D_Sy_* (m^2^ s^−1^)	*K_cy_* (m s^−1^)	*D_Sβ_* (m^2^ s^−1^)	*K_cβ_* (m s^−1^)	*K*	*Bi*	*RMSE*	*R* ^2^
Low	Low	Low	4.68 × 10^−12^	1.89 × 10^−4^	6.04 × 10^−14^	7.09 × 10^−11^	4.01 × 10^−2^	2.18 × 10^4^	0.955	0.978
Low	Low	High	2.76 × 10^−12^	1.20 × 10^−4^	1.29 × 10^−13^	1.51 × 10^−10^	9.95 × 10^−2^	1.62 × 10^4^	1.073	0.990
Low	High	Low	2.14 × 10^−12^	9.79 × 10^−4^	5.67 × 10^−14^	6.65 × 10^−11^	4.01 × 10^−2^	1.20 × 10^4^	0.765	0.984
Low	High	High	1.17 × 10^−12^	5.84 × 10^−4^	8.22 × 10^−14^	9.64 × 10^−11^	9.17 × 10^−2^	1.13 × 10^4^	0.748	0.995
Mid	Mid	Mid	3.58 × 10^−12^	1.48 × 10^−4^	1.26 × 10^−13^	1.47 × 10^−10^	8.84 × 10^−2^	1.81 × 10^4^	1.346	0.982
High	Low	Low	8.29 × 10^−12^	3.00 × 10^−4^	3.51 × 10^−13^	4.12 × 10^−10^	8.25 × 10^−2^	1.23 × 10^4^	2.685	0.925
High	Low	High	5.71 × 10^−12^	2.18 × 10^−4^	7.55 × 10^−13^	8.85 × 10^−10^	1.41 × 10^−1^	7.05 × 10^3^	1.062	0.993
High	High	Low	4.26 × 10^−12^	1.71 × 10^−4^	2.71 × 10^−13^	3.18 × 10^−10^	9.24 × 10^−2^	1.01 × 10^4^	1.592	0.978
High	High	High	2.77 × 10^−12^	1.18 × 10^−4^	6.43 × 10^−13^	7.54 × 10^−10^	2.22 × 10^−1^	7.09 × 10^3^	1.674	0.988

Full factorial analysis of variance (ANOVA) was conducted for experimentally determined parameters of solid-phase (internal) diffusivity ($D_{s\beta }$), solid phase (internal) mass transfer coefficient ($k_{c\beta }$), and distribution constant (K), with factor effects and associated statistical significance shown in Table 2.

**Table 2:** Factor effects and statistical significance of experimentally determined model parameters: solid-phase (internal) diffusivity ($D_{s\beta }$), solid phase (internal) mass transfer coefficient ($k_{c\beta }$) and distribution constant (K)

	*D_Sβ_*		*K_cβ_*		*K*	
Coefficient	Factor Effect	*p* Value	Factor Effect	*p* Value	Factor Effect	*p* Value
Temp. (A)	4.29 × 10^−13^	1.81 × 10^−5^ ***	5.03 × 10^−10^	1.81 × 10^−5^ ***	6.70 × 10^−2^	3.56 × 10^−6^ ***
Glucose (B)	−6.58 × 10^−14^	2.03 × 10^−1^	−7.71 × 10^−11^	2.03 × 10^−1^	2.06 × 10^−2^	8.64 × 10^−3^ **
Ethanol (C)	2.17 × 10^−13^	1.84 × 10^−3^ **	2.54 × 10^−10^	1.84 × 10^−3^ **	7.49 × 10^−2^	1.53 × 10^−6^ ***
AB	−4.09 × 10^−14^	4.14 × 10^−1^	−4.80 × 10^−11^	4.14 × 10^−1^	2.45 × 10^−2^	3.39 × 10^−3^ **
AC	1.70 × 10^−13^	7.28 × 10^−3^ **	1.99 × 10^−10^	7.28 × 10^−3^ **	1.93 × 10^−2^	1.19 × 10^−2^ *
BC	−1.77 × 10^−14^	7.18 × 10^−1^	−2.08 × 10^−11^	7.18 × 10^−1^	1.58 × 10^−2^	2.91 × 10^−2^ *
ABC	3.35 × 10^−15^	9.45 × 10^−1^	3.94 × 10^−12^	9.45 × 10^−1^	1.98 × 10^−2^	1.07 × 10^−2^ *

*, ** and *** represent factors that are statistically significant at the 5%, 1% and 0.1% levels, respectively.

**Table 3:** Summary of shape variables and physical properties of the system.

Property or Shape Variable	Value	Source
a (m^2^ m^–3^)	5747	Mathematically derived
ε	0.9173	Experimentally determined
$c_{\beta 0}$ (kg m^–3^)	6.987 × 10^–1^	Experimentally determined
L (m)	1.74 × 10^–4^	Jin et al. [24]
$V_{A}$ (L mol^–1^)	0.5259	Geankoplis [22]
$\mu_{\gamma }$ (cP)	Varied	Experimentally determined
$\rho_{\gamma }$ (kg m^–3^)	Varied	HYSYS (Hysys, Operations Guide., 2005)
$M_{\gamma }$ (g mol^–1^)	Varied	Equation (19)
φ	Varied	Equation (20)

## Supplementary Materials

Supplementary Materials are available online.

## Appendix A

a	Specific surface area for mass transfer (m^2^ m^−3^)
$b_{1},\ldots ,b_{4}$	Constants required to solve Equations (14) and (15)
c	Phenolic compound concentration (g m^−3^)
$C_{1},\ldots ,C_{4}$	Constants required to solve Equations (14) and (15)
$C_{EtOH}$	Ethanol concentration (% *v*/*v*)
$C_{g}$	Glucose concentration (g L^−1^)
D	Mechanical stirrer diameter (m)
$D_{s}$	Solute (s) mass diffusivity (m^2^ s^−1^)
$k_{c}$	Mass transfer coefficient (m s^−1^)
K	Distribution constant
L	Characteristic length for diffusive mass transfer (m)
M	Molar mass (g mol^−1^)
N	Stirrer speed (rpm)
$r_{1,2}$	Constants required to solve Equations (14) and (15)
$R^{2}$	Coefficient of determination
RMSE	Root mean square error
t	Time (s)
T	Temperature (°C)
$V_{A}$	Solute molar volume (L mol^−1^)

## Appendix B

Bi	Biot number
Re	Reynolds number
Sc	Schmidt number
Sh	Sherwood number

## Appendix C

ε	Solvent volume fraction
φ	Association parameter
μ	Dynamic viscosity (kg m^−1^ s^−1^)
ρ	Density (kg m^−3^)
$\psi_{1,2}$	Constants required to solve Equations (14) and (15)

## Appendix D

0	At the initial or reference point
e	At the equilibrium stage
exp	Experimentally obtained
i	At the solid-liquid interface
pred	Predicted by model
β	Solid phase
γ	Liquid phase
